# PROTOCOL: Impact of the food environment on diet‐related health outcomes in school‐age children and adolescents in low‐ and middle‐income countries: a systematic review

**DOI:** 10.1002/CL2.198

**Published:** 2018-10-12

**Authors:** Bianca Carducci, Christina Oh, Emily C Keats, Michelle F Gaffey, Daniel E Roth, Zulfiqar A Bhutta

## Background

### The problem, condition or issue

Current estimates indicate that some form of malnutrition (undernutrition, overweight or obesity, and poor dietary habits) affects one in three people worldwide (International Food Policy Research Institute, 2016), and the 2016 Global Burden of Disease study has placed poor dietary habits as one of the leading risk factors for mortality globally (Collaborators GBDRF, 2017). Over the past decade, there has been great momentum around addressing malnutrition in all its forms and commitment to actions that can accelerate progress to reduce its associated burden of morbidity and mortality. In 2012, the World Health Assembly (WHA) adopted the 2025 Global Targets for Maternal, Infant and Young Child Nutrition and in 2013, WHA adopted targets for non‐communicable diseases (NCDs), including several nutrition‐relevant targets (International Food Policy Research Institute, 2016). More recently, the United Nations elevated its efforts through a global declaration of 17 Sustainable Development Goals (SDGs), where at least 12 of the 17 goals feature indicators relevant to nutrition. In line with these targets, the decade of 2016‐2025 has been declared the Decade of Action on Nutrition (International Food Policy Research Institute, 2016). To this end, prioritizing critical actions to address school‐age children and adolescent nutrition, is necessary to achieve these milestones. At the forefront of malnutrition and poor dietary intake is the food system.

#### The food system

According to the Food and Agriculture Organization (FAO) High Level Panel of Global Food and Nutrition Security, the food system is defined as ‘a system that embraces all the elements (environment, people, inputs, processes, infrastructure, institutions, markets and trade) and activities that relate to the production, processing, distribution and marketing, preparation and consumption of food and the outputs of these activities, including socio‐economic and environmental outcomes' (High Level Panel of Experts, 2017). Importantly, this group identified three food system typologies (i.e. traditional, mixed and modern), based on distinct inputs (natural resources, human capital, physical capital, agriculture and food technology), outputs (food purchasing patterns, diet, health and environmental sustainability) and processes (food production, supply chains and the food environment) Table 1. The transition from traditional to industrial food systems has been linked to urbanization, policy liberalization, agricultural productivity and income growth. In addition, the Global Nutrition Report, (International Food Policy Research Institute, 2015) defined two additional food system typologies (emerging and transitioning food systems), which are variations of the mixed food system, often observed in low‐ and middle‐income countries (LMICs). Importantly, multiple types of food systems, and their associated food supply chains and food environments can co‐exist within a single country simultaneously.

**Table 1:** Food system typologies and their food supply chains and food environments

**Food supply chains**	**Traditional food systems**	**Mixed food systems**	**Modern food systems**
***Production (availability)***	Food is mainly produced by smallholders in the area and most of the foods available are local and seasonal.	Food production takes place at both local smallholder farms and larger farms that are farther away. There is greater access to foods outside their typical season.	A wide array of foods is produced at farms ranging from small to industrial in size. Production is global, so foods are available from anywhere and at any time.
***Storage and distribution***	Lack of adequate roads makes transporting food difficult and slow, leading to food waste. Poor storage facilities and lack of cold storage makes storing food, especially perishables, difficult and leads to food safety concerns and waste.	There are improvements in infrastructure with better roads, storage facilities and increased access to cold storage; however, these are usually not equally accessible, especially for the rural poor.	Modern roads, storage facilities and cold storage make it easy to transport food on long distances and store it safely for long periods of time.
***Processing and packaging***	Basic processing is available such as drying fruit, milling flour or processing dairy. Little or limited packaging occurs.	Highly‐processed packaged foods emerge and are more accessible. These extend the shelf life of foods.	Many processed packaged foods are easily available, often cheap and convenient to eat, but sometimes “unhealthy”.
***Availability and physical access (proximity)***	Higher density of local informal markets but longer distances to access formal markets and poor or non‐existent roads make travel difficult and long.	There is still a high density of informal markets but there is also a larger number of formal markets. Better road and vehicle access emerges, increasing consumer access to different foods. However, low‐income consumers often have less access to transportation.	Reliance is on formal markets with locations in close proximity with easy accessibility. Low‐income areas can often be qualified as food deserts or food swamps.
***Economic access (affordability)***	Food is a large portion of the household budget. Staples tend to be significantly less expensive relative to ASF, which tend to be more expensive.	Food places moderate demands on the household budget. Staples are inexpensive, whereas ASF and perishable foods are expensive. Many highly processed and convenience foods are inexpensive.	Food demands less of the household budget. The price of staples is lower relative to ASF and perishable foods, but the difference is less stark than in the other systems. With more options, specialty items (e.g. organic, locally produced) tend to be more expensive.
***Promotion, advertising and information***	Very little promotion, with the exception of the efforts of some multi‐national companies. Posters, signs in kiosks and on buildings, some billboards. Very little information in terms of labelling and guidelines. Information disseminated largely through public health nutrition education.	Branding and advertisements become more common, including on billboards, print, radio, television and the Internet. Some information provided, and labels on food products and on the shelves of stores. Dietary guidelines available, but with little or no access in some areas.	High level of food promotion via multiple media channels. Marketing targeted to specific groups (e.g. children). High level of information on labels, shelves in stores and menus. High level of information from public health campaigns.
***Food quality and safety***	Low control of quality and food safety standards. Little to no cold storage. Less of a demand for quality ingredients.	Quality and food safety controls exist, but are often not adhered to. Food safety adherence is often limited to branded processed packaged foods. Cold storage exists, but is not reliable. Ingredient lists on foods but less emphasis on “natural” or “organic.”	Food safety standards are closely adhered to and monitored. Cold storage is prevalent and reliable. Ingredients listed and standardized. Demand for foods and animals grown in certain ways adhering to sustainability and animal welfare practices.

Within traditional (or rural) food systems, there is a greater proportion of informal food markets (i.e. wet markets, mobile street vendors), compared to formal food outlets, as food is produced locally by rural smallholder farmers, is minimally processed and dependent on seasons. Moreover, there is minimal food promotion through advertising, limited food quality control and increase food affordability as a significant portion of monthly household expenditure is allocated to food. Examples of countries with such food system type are: Bangladesh, Ethiopia, Indonesia, Nepal, Senegal and Zimbabwe.

Emerging food systems are characterized by low urbanization and agricultural productivity, though there is still reliance on staple foods and household food budgets are considered moderate to high. Examples of countries with such food system type are: Cameroon, China, Honduras, Pakistan, Namibia, Philippines and Thailand.

In transitioning food systems, there is a shift towards moderate agricultural productivity and lower dependence on staples as compared to emerging food systems. As well, household food budgets are still considered high compared to mixed food systems. Country examples from this food system type are: Brazil, Ecuador, Guyana, Malaysia, Mauritius, Russia, Suriname and Ukraine.

In mixed food systems, there is improved infrastructure to support formal markets, though informal markets are still prominent. Furthermore, there is increased diversity of foods available, including staples, fresh produce, animal‐sourced foods and highly‐processed packaged foods. There is increased food promotion through advertisements and weakly implemented regulations on food safety and quality. Examples of countries with such food system type are: Barbados, Bulgaria, Estonia, Germany, Hungary, Italy and Switzerland.

In modern (or industrial) food systems, the highly urbanized environment promotes increased access and reliance on highly diverse and dense formal food markets (i.e. large supermarkets, hypermarkets, fast‐food and fine dining restaurants). A high level of food information and promotion exists through various media platforms, including marketing and labelling on food packages, advertisements and public health campaigns. Economically, household food expenditure is reduced, though fresh produce and animal‐sourced foods are more expensive than staples and packaged products. Examples of countries with such food system type are: Australia, Canada, Denmark, Lebanon, Republic of Korea, Sweden and the US.

#### The food environment

In 2012, a global network of public‐interest organisations and researchers aiming to monitor, benchmark and support public and private sector actions to create healthy food environments and reduce obesity and NCDs was established. This network, named The International Network for Food and Obesity/Non‐Communicable Diseases Research, Monitoring and Action Support (INFORMAS), defines the food environment as ‘the collective physical, economic, policy and sociocultural surroundings, opportunities and conditions that influence people's food and beverage choices and nutritional status' (Swinburn et al., 2013). In the same light, the FAO High Level Panel of Experts for Food Security and Nutrition characterizes the food environment as ‘food entry points’ (i.e. the physical spaces where food is obtained; the built environment that allows consumers to access these spaces); personal determinants of food choices (including income, education, values, skills, etc.); and the political, social and cultural norms that underlie these interactions. The key elements of the food environment that influence food choices, food acceptability and diets are: physical and economic access to food (proximity and affordability); food promotion, advertising and information; and food quality and safety (High Level Panel of Experts, 2017).

It is important to note that there are several proposed conceptual frameworks with a global lens available from Health Canada, Centre for Disease Control (CDC), USA, the FAO High Level Panel of Experts for Food Security and Nutrition, and the Global Panel on Agriculture and Food Systems for Nutrition (Health Canada, 2013; CDC, 2013; Development Initiatives, 2017; High Level Panel of Experts, 2017; Global Panel on Agriculture and Food Systems, 2016), which borrow from the early work of Penchansky and Thomas (1981). These food environment models have been conceptualised to possess five dimensions that impact food choice and dietary intake: availability, spatial accessibility, affordability, accommodation and acceptability. Others have proposed a food environment model with four domains: community, consumer, organisational and information (Glanz, Sallis, Saelens and Frank, 2005) or two domains: the external food environment and the personal food environment (Turner et al., 2017). Importantly, this latter model differs from previous models as it aims to position exogenous dimensions at a structural level (i.e. vendor and product properties, marketing and regulation, availability and prices) with endogenous dimensions (i.e. accessibility, affordability, convenience and desirability) at the individual level.

Though these models have laid the foundation within the food environment space, none are tailored to the complexity of LMICs or are specific to school‐age children and adolescents. Rapid globalisation and urbanisation in LMICs has incited major changes to the landscape of food. This is observed upstream with trade liberalization and foreign direct investments by transnational food and beverage companies, including grocery and fast food retailers. As well, transformations in food processing and modernised supply chains have caused a systemic shift in the composition and packaging of foods, as well as the ease of acquiring these foods. In parallel, traditional domestic channels of food acquisition, such as through informal unregistered vendors (wet markets and street vendors) still account for a portion of the market share in LMICs. This dynamic and opportunistic environment is in a constant flux, posing a challenge in accurately estimating food availability, accessibility and affordability. Downstream, food preferences, dietary patterns, and habits have been negatively impacted as economical, convenience foods are inexpensive compared to healthy options. For school‐age children and adolescents, this corresponds to increased reliance on unhealthy, nutrient‐poor ultra‐processed foods and sugar‐sweetened beverages, as well as, increased snacking and eating away‐from‐home. Taken together, energy imbalances have led to population‐wide nutrition transition with continued burden of stunting and wasting. This double burden of malnutrition has been widely studied in LMICs, though there have been insufficient efforts to study the association to the food environment (Popkin and Reardon, 2018; Baker and Friel, 2016; International Food Policy Research Institute, 2017).

**Figure 1:**
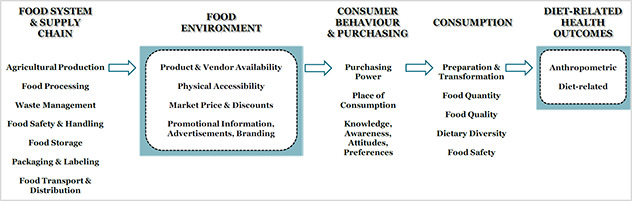
Conceptual Framework of the Food Environment in School‐age Children and Adolescents in LMICs

Our conceptual framework aims to fill the current gap, by focusing on extrinsic factors within the food environment that influence school‐age children and adolescents in LMICs. Importantly, Figure 1 is not a definitive model of existing evidence. Rather, it is a proposed logic model to help guide the implementation of this review. It is believed that the food environment is a complex adaptive system, influenced by the wider food system, whereby various industries and actors operate interdependently and adaptively, and their interaction is often shaped through spatial and temporal complexity. Though others have depicted specific micro‐food environments (i.e. home, school, consumer food environments or external versus personal food environments), we are not attempting to categorise these micro‐food environments. We acknowledge these micro‐food environments exist and interact with one another, especially in terms of the translation of food and nutrition knowledge, perceptions and desirability. For example, education on healthy food choices learned in the school food environment would influence an adolescent in the market food environment.

As seen in Figure 1, it is believed that the food environment innately possesses the following dimensions:

Accessibility: This refers to proximity, density and presence of retailers relative to individuals or organisations. Availability: This refers to both retailers and product availability within a given context. Pricing: From a market perspective, this is indicative of the market price of products. From a household or individual perspective, this equates to affordability (purchasing power). Our review will focus on market price, as this is most relevant to environment level interventions. Promotion: Promotional advertising, marketing and branding directed at individuals.

These dimensions and their associated measures are useful for understanding the food environment, especially in urbanised settings of LMICs. As well, the intersection of these dimensions directly or indirectly involves primary and secondary sectors such as agriculture, food manufacturing and food processing. We recognise policy is an important component of the food environment, however, we have not included policy as its own dimension. This is because we believe policies, regulations and guidelines (i.e. national, regional, local) underlie all dimensions of the food environment. Likewise, we also acknowledge that conceptually food quality and food safety (including food handling, sanitation and hygiene) are integral components of the food environment. However, food safety and food quality are in a constant flux, making it difficult to establish metrics of the food environment, particularly for school‐age children and adolescents. For this reason, we have chosen to place food quality and food safety under the food system and supply chain in our model (Figure 1).

Additionally, it is hypothesised that the food environment is linked to consumer behaviour and purchasing, which includes knowledge, awareness, attitudes and preferences. As seen in Figure 1, consumer behaviour and purchasing are extrinsic to the food environment, and subsequently affect consumption (preparation & transformation, quantity, quality, diversity and safety) and ultimately, diet‐related health outcomes in school‐age children and adolescents in LMICs.

We acknowledge the limitation in developing a single conceptual framework for both school‐age children and adolescents. School‐age children differ from adolescents in that, school‐age children have limited autonomy in the acquisition of food. Instead, parents and caretakers influence their consumption by making decisions on what is purchased for the home, what is eaten outside of the home and setting limits on the quantity of food consumed. On the other hand, adolescents have the ability to make their own decisions and purchase on their own. However, the focus of our review is to understand the impact of food environment interventions on diet‐related health outcomes in school‐age children and adolescents, where food purchasing is beyond the scope of what we have considered to be intrinsic properties of the food environment, whether this is done by parents, providers or adolescents.

### The intervention

With global increases in obesity prevalence, especially in LMICs, there are various interventions that have been categorised and implemented with intent to improve the food environment (Lobstein et al., 2015). These interventions are often best evaluated in randomised controlled trials (RCTs) whereby the efficacy and degree to which interventions produce an impact of change are tested under optimally controlled conditions, minimizing bias and confounding factors (Marchand, Stice, Rohde and Becker, 2011). However, there are interventions (e.g. policy‐based or food‐retail interventions) that cannot be so rigorously randomised and controlled, and as such, the RCT is not an appropriate study design. In these cases, interrupted‐time‐series (ITS) and quasi‐experimental designs are used for impact evaluation (Taillie et al., 2017).

ITS and quasi‐experimental studies evaluate a change or exposure that is outside the control of the researchers, proving more difficult to manipulate the exposure and randomise participants into intervention and control groups (Craig et al., 2012). These limitations present several challenges, such as ensuring strong internal and external validity, minimizing potential unmeasured confounders, mitigating pre‐existing baseline differences in the outcome between groups, and being flexible with uncontrolled timing and seasonality, etc. Despite this, ITS and quasi‐experimental studies offer greater generalisability than RCTs. As well, they provide critical insight and information about real‐world settings, such as how feasible the implementation of an intervention is in situations where a considerable lack of control exists (Taillie et al., 2017).

In addition, food environment interventions can be classified as preventive or management‐based (Swinburn and Egger, 2002; Harvard, 2017). Preventive interventions intend to improve the food environment by minimizing an individual's exposure to unhealthy environments (e.g. limiting unhealthy food options in school cafeterias) or by preventing and mitigating poor health with proactive strategies (e.g. with programs targeting increased physical activity and minimized television or ‘sitting time’) (Harvard, 2017). Management‐based interventions involve long‐term strategies to improve population health, such as reducing the population's prevalence of obesity. Examples of these interventions in school‐age children and adolescents include public education campaigns and policy interventions on taxation and television advertising of unhealthy foods (Swinburn and Egger, 2002).

Interventions can also be defined as environmental or behavioural (Roberto et al., 2015; World Cancer Research Fund, 2017), whereby environmental interventions target the various spheres of an individual's environment that influence their choice in food and beverages. These include the built and natural physical environments, legal and political environments, socio‐economic, and cultural environments. Examples of environmental interventions specific to school‐age children and adolescents include school‐feeding and school‐meal programs or policies that incentivise vendors to provide healthier retail environments. Behavioural interventions place a greater focus on the individual, targeting their knowledge, attitudes, perceptions, preferences, and abilities in food and beverage choice and consumption patterns. Several theories and models of behavioural change such as the theory of planned behaviour, diffusion of innovation theory, the social cognitive theory, and the health belief model, offer insight into the behavioural relationship between food environment interventions and diet‐related health outcomes. Behavioural interventions targeting school‐age children and adolescents include nutrition counselling and education for educators and students, physical activity programs, and public awareness campaigns (World Cancer Research Fund, 2017). It is acknowledged that overlap in interventional effects may occur whereby interventions, such as nutritional labelling policies, may alter both the environment and behaviour of the individual or consumer. Likewise, there may be instances where interventions could be categorised as both preventive (and or management‐based), as well as environmental (and or behavioural).

This behavioural‐environmental approach is consistent with other models and frameworks designed to identify and categorise distinct areas of policy and intervention actions. Similarly, the NOURISHING framework, created by the World Cancer Research Fund International (WCRFI) identifies ten specific areas where policy and interventions can be used to improve food environments (World Cancer Research Fund, 2017; Hawkes, Jewell and Allen, 2013). While designed to be globally applicable, WCRFI recognises that the framework's key areas would need to be adapted to the specific context and population of different countries.

Further, interventions can be classified by their level of implementation. Swinburn and Egger, 2002 suggest that interventions can be categorised into two main groups. A ‘settings‐based’ intervention would focus primarily on micro‐environments (micro‐level) such as schools, workplaces and neighbourhoods, whereas ‘sector‐based’ interventions would target macro‐ and meso‐level environments such as the food industry and national supply chain (Swinburn and Egger, 2002). A simplified logic model from the CDC distinguishes four levels of implementation: policy‐level, community‐level, organisational‐level and the individual‐level. The policy‐level includes interventions that influence legislation and policies that have a macro‐level exposure, such as food labelling laws and supply chain regulation. Community‐level interventions focus on increasing the awareness of the general population, while the organisational‐level interventions are intended to influence organisations and systems such as health‐care systems, industry players and community‐based organisations. Lastly, the individual‐level defines interventions as those that improve and enhance the knowledge, skills, attitudes, abilities and preferences of the individual (CDC, 2013).

Specific to our review, school‐aged children and adolescent engage with the food environment in various ways. They interact through micro‐food environments such as the home, school and workplace, and consumer and retail spheres. While our review does not concern interventions implemented at the household level, we acknowledge that dietary intake in school‐aged children and adolescents is affected by factors in which they have limited control. Adolescents, but especially children, may not have full autonomy and choice over what is made available to them and what they consume. These include: what is available in their homes, what their parents or providers purchase, and what the household can afford.

The school and workplace can be important food environments for children and adolescents. At school and work, their food choices and consumption are influenced by what is available and accessible (e.g. in cafeterias, vending machines, food stalls on or near campus). Thus, organisational‐level interventions that change the physical environment such as improving the quality of foods available at lunchtime could have an impact on the dietary intake and consumption, and thus health outcomes, of school aged children and adolescents.

We recognise also that not all children and adolescents attend school, especially in LMICs. However, we aim to capture these children and adolescents through community‐level interventions or organisational‐level interventions specific to the workplace (for those children and adolescents who are in the workforce). One limitation we acknowledge is that some children and adolescents who do not attend school might be better captured at the household level. However, the food environment at the household level is out of this review's scope as it can be affected by other factors, such as provider's preference and household income, which are not specific to the child or adolescent him‐ or herself.

**Figure 2:**
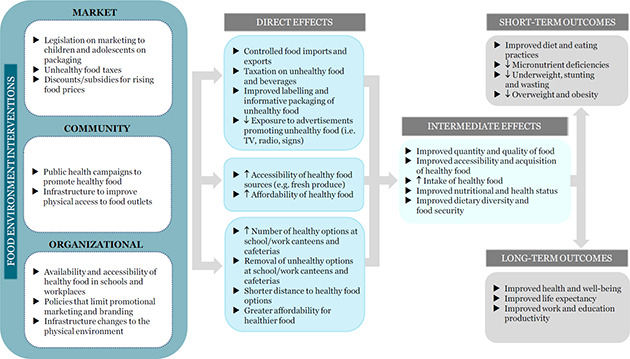
Impact of Food Environment Interventions on Diet‐related Health Outcomes in School‐age Children and Adolescents in LMICs

This review will include environmental interventions, both preventive and management‐based, targeted at school‐age children and adolescents in LMICs, which are implemented through market, community and organisational platforms (Figure 2). For the purposes of this review, we will consider any policy, regulation or guideline that affects the food environment through each of the three platforms, as an intervention. For more details on specific intervention types, please see the section “Types of interventions”.

### How the intervention might work

Today, many food environments promote access to unhealthy foods, influencing individuals' preferences and demands for foods with poor nutritional quality. This has contributed to the rise in obesity and non‐communicable diseases globally, especially in low‐ and middle‐income countries (Roberto et al., 2015). In response, food environment interventions are designed and implemented to improve these environments, which ultimately interact with other factors, affecting people's food and beverage perceptions, choices and intake. Many frameworks and models have conceptualised this complex relationship between the individual and the host of environmental and health factors that contribute to his/her dietary behaviour and nutritional status (Health Canada, 2013; Development Initiatives, 2017; High Level Panel of Experts, 2017). The intended effects of these interventions are thus to change and improve nutritional intake and dietary behaviour in a global effort to reduce the rise in obesity and non‐communicable diseases. To illustrate these effects, we developed a logic model that depicts how different types of interventions, at various levels of implementation and influence, address food and nutrition insecurity and thus, affect the diet and health‐related outcomes at the individual level.

Environmental interventions, such as those categorised in the NOURISHING framework, would primarily work at the policy and community levels of implementation within the CDC's logic model, or at the macro‐level as defined by Swinburn (2002) (CDC, 2013).

In our model, policies, regulations or guidelines at every level (i.e. national, regional and local) affect the food environment through all three platforms (market, community and organisational). These include macro‐level legislation on taxation of unhealthy food, regulation of imports and exports, and policies on nutritional labelling and advertising, each of which would have an effect on the economy, various industries and society at‐large. The direct effects of these interventions would include food price regulation, taxation on unhealthy food and beverage, improved labelling and informative food packaging, and decreased airtime for TV advertisements of unhealthy food during children's programs. Examples of micro‐level legislation or policy would include school‐based policies that limit the selling of sugar‐sweetened beverages in a school cafeteria.

Similar to other models and frameworks cited above, market‐level interventions in our logic model include those that affect change within the formal and informal retail space (i.e. changing the placement of products within a store, changing the diversity of products available for purchase or store‐directed discounts on healthy food.) Community‐level interventions affect widespread changes in physical infrastructure within the community and public health. These interventions include public health campaigns promoting healthy food and nutrition, transportation infrastructure improvements to provide better access and availability of healthy food options. Direct effects of these interventions include increasing the number of food outlets in the neighbourhood and providing subsidies for healthier options in stores. At the organisational‐level, interventions aim to influence institutions, such as work and school systems. These involve regulating the availability of food in work and school environments, such as increasing the number of healthy options at school canteens and cafeterias, and improving the physical environment, such as the implementation of water fountains in schools and removing advertisements (Figure 2).

The combination of these direct effects from the market‐, community‐ and organisational‐level interventions work to improve the acquisition of healthier food and beverages for both households and individuals, in terms of both quantity and quality. Better availability and accessibility to healthy food and beverages allows children, adolescents and their families to make healthier food choices and improve their food intake. These intermediate effects lead to short‐term outcomes, such as improved diet and eating practices, reduced overweight and obesity, and long‐term outcomes, such as improved life expectancy and improved work and education productivity.

As mentioned above, we acknowledge that school‐aged children and adolescents may experience the food environment differently as they transition from child to adolescent, or adolescent to adult. As children get older, they gain independence, autonomy and a greater range of choice in their acquisition of food and drink. This would affect their consumption and subsequently, their health outcomes. We also acknowledge that different environments might provide greater autonomy and choice. For example, a child eating lunch at school might have unhealthier options provided to them than at home where their parents are able to better control their diets. However, the focus of our review is to understand the impact of interventions outside of the home environment, apart from the strong influence that providers and caretakers might have on the diets of children and adolescents.

### Why it is important to do the review

Understanding if and how food environment interventions impact diet‐related health outcomes in school‐age children and adolescents in LMICs is critical for offering strategic recommendations for food environment improvement and encourages advocacy. Ultimately, this review will contribute to research within this field, with the overarching goal to improve health outcomes through recommended interventions. As mentioned, food environments in LMICs differ from those in high‐income countries in part due to the lack of appropriate infrastructure to access and distribute healthy foods. The lack of registered and formal outlets complicates measuring food environments in LMICs through geospatial and observational methodologies. As such, we acknowledge there are limitations associated with synthesizing information on food environments in LMICs, as food environments are not static. Despite this, we will attempt to capture information on the transitory nature of the food environment.

To date, there is paucity of high‐quality summative evidence to link the food environment to diet‐related health outcomes in school‐age children and adolescents in LMICs. In fact, a lack of consistency in the definition of a food environment has presented challenges in synthesizing data. Currently, the only attempt to develop standard indicators of the food environment, relates to policy indicators. The Healthy Food Environment Policy Index (Food‐EPI), was developed by the INFORMAS group to assess the level of policy implementation against international best practices. Food‐EPI consists of seven domains in relation to policy (food composition, labelling, marketing, provision, retail, prices and trade) and six domains in relation to infrastructure support (leadership, governance, funding and resources, monitoring and intelligence, platforms for interaction and health‐in‐all‐policies). Currently, Food‐EPI is being conducted in 30 countries worldwide, of which half are in progress in LMICs. To our knowledge, there are no systematic reviews on this topic that have been conducted or are in progress on LMIC populations.

Related literature and systematic reviews have been focused in high‐income countries. These reviews have examined:

► Food environment methodology (McKinnon et al., 2009; Glanz, 2009; Charreire et al., 2010; Kelly, Floor and Yeatman, 2011; Gustafson, Hankins and Jilcott, 2012; Caspi, Sorensen, Subramanian and Kawachi, 2012; Lytle and Sokol, 2017);
► Food environment interventions (Black, D'Onise, McDermott, Vally and O'Dea, 2017; Townshend and Lake, 2017; Penney et al., 2015);
► Food environment intervention cost‐effectiveness (McKinnon et al., 2016);
► Food environment exposures in association with dietary intake, dietary behaviour and or diet‐related health outcomes (Holsten, 2009; Cassagrande et al., 2009; Dunton, Kaplan, Wolch, Jerrett and Reynolds, 2009; Feng, Glass, Curriero, Stewart and Schwartz, 2010; Osei‐Assibey et al., 2012; Williams et al., 2013; Kirkpatrick et al., 2014; Driessen et al., 2014; Engler‐Stringer, Le, Gerrard and Muhajarine, 2014; Williams et al., 2014; Martin, Ogilvie and Duhrcke, 2014; Gamba, Schuchter, Rutt and Seto, 2015; Lenardson, Hansen and Hartley, 2015; Roy, Kelly, Rangan and Allman‐Farinelli, 2015; Cetateanu and Jones, 2016; Cobb et al., 2015; Pineda and Mindell, 2016; Olstad et al., 2017).

Three on‐going reviews (registered with PROSPERO) are also focused in high‐income countries (Pacheco, Balan, Archibald, Grant and Skafida, 2017; Sisnowski, Street and Merlin, 2015; Almiron‐Roig, Beasley, Stevens, Bryant, Kirk, 2014).

It is also worth noting the Cochrane childhood obesity intervention reviews. A total of seven reviews have been published, one prevention‐focused (Waters et al., 2011) and six which are management‐focused through six approaches including surgery, drugs, parent‐only interventions, diet, physical activity, and behavioural interventions for young children (aged 0 to 6 years), schoolchildren (aged 6 to 11 years) and adolescents (aged 12 to 17 years) (Mead et al., 2017; Mead et al., 2016; Colquitt et al., 2016; Al‐Khudairy et al., 2017; Loveman et al., 2015; Ells et al., 2015). Our review differs from the three relevant Cochrane reviews (Waters et al., 2011; Mead et al., 2017 and Al‐Khudairy et al., 2017) in that we will include both preventive and management‐based behavioural and environmental interventions at the food environment level for multiple diet‐related health outcomes including, but not limited to, obesity.

Moreover, our inclusion criteria contrasts to that of the Cochrane reviews in that we do not have a minimum intervention time, and we will not include dietary intake interventions alone or physical activity interventions alone (i.e. a dietary intake only or physical activity only intervention must be in combination with a food environment intervention to be eligible). This is because the focus of our review aims to understand the impact of interventions on the physical environment, to which school‐age children and adolescents are exposed. Whereas, dietary intake or physical activity interventions serve to modify individual behaviour (i.e. changing their eating patterns or activity level). Finally, for all three reviews, the majority of included studies were conducted in high‐income settings, whereas our review is focused in LMIC settings.

## Objectives

Our main objective for this review is to synthesise and assess literature on the impact of food environment interventions including interventions that attempt to influence food availability, accessibility, policy, pricing and promotion on diet‐related health outcomes in school‐age children and adolescents in LMICs, published since 2000.

## Methodology

### Criteria for including and excluding studies

#### Types of study designs

We will consider the following primary study types, including large‐scale programme evaluations, which assess the efficacy and/or effectiveness of interventions:

1. Randomised controlled trials (RCTs): Experimental studies in which people are allocated to different interventions using methods that are random. This includes both individually‐randomised controlled trials, where the randomisation occurs on the level of individuals, and cluster‐randomised controlled trials (cRCTs), in which the randomisation occurs on the level of clusters of individuals (i.e. study groups or study sites). An example of this type of study would be the effect of promotional materials on overweight/obesity status. Children would be randomised by schools within a district, where the experiment group would have vending machines and promotional materials (posters on food/beverage products), while the control group would not have vending machines and promotional materials;
2. Non‐randomised controlled trials (NRCTs) or quasi‐experimental: Experimental studies in which people (individuals or clusters of individuals) are allocated to the intervention or control group using methods that are not random, including exogenous geographical variation in the treatment allocation (natural experiments); An example of this type of study would measure anthropometric outcomes of school‐age children and adolescents within schools that are < 400 meter radius to a food outlet/vendor, compared to those within schools that are > 400 meter radius to a food outlet/vendor. Children within each comparison group are not randomised, though are allocated to the exposure based on geographical variation;
3. Controlled before‐after (CBA) studies: Studies in which observations are made before and after the implementation of an intervention, both in a group that receives the intervention and in a control group that does not. Unlike in NRCTs, the allocation to the intervention and control groups is not determined by the investigators, but by nature or by other factors outside the control of the investigators. An example of this type of study would be anthropometric measurements of all school‐age children and adolescents are taken before and after the intervention of increased healthy food availability within a school cafeteria. The children's' *before* measurements act as control to the after measurements once the intervention was implemented;
4. Interrupted‐time‐series studies: Studies that use observations at multiple time points before and after an intervention (the ‘interruption’). The design attempts to detect whether the intervention has had an effect greater than any underlying trend over time. An example of this type of study would be implementation of a region‐wide sugar tax on unhealthy food products. Anthropometric measurements of school‐age children and adolescents within this region would be taken three time points before and three time points after the implementation of this tax.
5. Repeated measures studies (RMS): An ITS study where measurements are made in the same individuals at each time point.

For the exact classification of study designs we will use the algorithm provided by EPOC, 2013.

We will include only study designs that satisfy the following criteria specified by EPOC, 2013:

► For cluster‐RCTs, NRCTs and CBA studies: studies with at least two intervention and two control sites
► For ITS and RMS: a clearly defined point in time when the intervention occurred and at least three data points before and three data points after the intervention.

In addition, we will include mixed methods studies (i.e. studies that include and report on both quantitative and qualitative findings). However, we will only include the quantitative aspects of these types of studies.

We will exclude animal studies, observational (quantitative and qualitative studies), dissertations, conference abstracts and other systematic reviews (though reference lists of relevant systematic reviews will be reviewed for studies that meet our eligibility criteria).

#### Types of participants

We will include participants aged 5‐9 years (school‐age children) and 10‐19.9 years (adolescents) from LMICs (Table 2). Children with conditions that could impact dietary intake or diet‐related health outcomes will be excluded (for example HIV, Tuberculosis, or growth disorders). Children and adolescents who are in school and out of school are eligible for inclusion. Studies that include participants outside of this age range (i.e. 20 to 24 years or 2‐4 years) and do not report disaggregated data will be excluded.

**Table 2:** PICO table

**Elements**	**Concepts**
**Population**	► Low‐ or middle‐income country, according to World Bank (for multi‐country studies that include high‐income countries, data must be disaggregated) ► School‐age children (5‐9.9 years) and or adolescents (10‐19.9 years), both males and females
**Intervention**	► Single component or multi‐component food environment interventions (preventive or management‐based) implemented through market, community or organisational platforms which relate to: ▷ Food availability ▷ Food accessibility (proximity or density) ▷ Food promotion (prominence within shelves/placement, social media or advertising, population nutrition promotion) ▷ Food policy, regulation and compliance (food labelling/packaging, sales, marketing, taxes) ▷ o Food pricing (affordability)
**Control**	Participants with no intervention, those who receive the standard of care or business as usual.
**Outcomes**	Primary outcomes: Anthropometric or Diet‐related Health Outcomes ► Incidence, prevalence or mean difference in: Height, weight, MUAC, stunting, wasting, BMI, age‐ and sex‐standardized body mass index (zBMI or BMI z‐scores), lean mass %, fat mass %, waist circumference, waist‐to‐hip ratio, overweight and obesity ► Incidence, prevalence or mean difference in: anaemia, type 2 diabetes, metabolic syndrome, goitre, night blindness

#### Types of interventions

This review will include environmental interventions, both preventive and management‐based, targeted at school‐age children and adolescents in LMICs, which are implemented through market, community and organisational platforms (Table 3), with the exception of those that are solely behaviour‐focused at an individual‐level (i.e. nutrition education/counselling), diet‐related at the individual‐level such as supplementation, calorie‐restriction, and intake of special food items (e.g. gluten‐free, organic, GMO, high‐fat, high‐sugar), and physical activity‐focused. These interventions do not fall within the scope of our review as our main focus is on interventions that pertain to the food environment. Moreover, there will be no minimum intervention time limit, as environmental interventions could be at a single time point (i.e. removal of vending machines from a school).

**Table 3:** Classification of food environment intervention areas

**Intervention Area**	Examples
**Market‐level**	
Availability	Interventions that increase the availability of healthy food options or limit availability of unhealthy food items at food stores
Policy	Policy interventions that implement unhealthy food taxes; policies on marketing on food products directed towards children
Pricing	Interventions that promote store‐directed price discounts on healthier food choices; point‐of‐sale monetary incentives for choosing healthier foods
Promotion	Interventions that limit food advertisements or media of food and beverages
**Community‐level**	
Accessibility	Interventions that addresses physical access to food outlets (i.e. transport and infrastructure)
Policy	Interventions that introduce policies on the reformulation of products to reduce sodium and trans‐fats; portion size limits; nutrient lists on food packages and menus; regulation on nutrient health claims; regulation of food advertising during peak hours of children's TV
Promotion	Health promotion campaigns by MOH to limit soda intake and replace with water; promotion of community gardens to increase fruit/vegetable availability
**Organisational‐level**	
Accessibility	Interventions that improve access by constructing a cafeteria/tuck shop within or near a school; installation of water fountains
Availability	Restricting sale of unhealthy food in cafeterias/tuck shops; increasing the availability of fruits and vegetables within the school; removal of vending machines within schools
Policy	Restriction of unhealthy vending machines in a school; restriction of food vendors in proximity to school
Promotion	Restriction of food advertising and other forms of commercial promotion of unhealthy diets within schools; promotion of water in place of sugar‐sweetened beverages

In regards to market‐level food environment interventions, we will include interventions within consumer retail environments, both informal and formal, (supermarkets, butchers, bakeries, cafes, restaurants, mobile food vendors, markets etc.) aimed at impacting diet‐related health outcomes (including overweight and obesity). Some examples include: policy interventions on taxation, marketing or labelling of unhealthy food products; pricing interventions where point‐of‐sale vouchers/coupons are given to encourage healthy food purchasing; increased healthy promotional material (advertisements, media) on healthy food and beverage; increased availability of healthy food and beverages/decreased availability of unhealthy food and beverages; increased access to healthy mobile food vendors).

In terms of community‐level food environment interventions, we will include studies where population policies or programs (i.e. public health campaigns to reduce food advertising during children's TV hours) have been implemented widely through households or the community.

At the organisational‐level, we will include food environment interventions implemented through schools or workplaces (i.e. school policies to limit food vendors in proximity to schools, improve accessibility and availability of healthy food options within and around the school) aimed at understanding the impact on diet‐related health outcomes (including overweight and obesity).

In regards to multi‐component interventions, we will consider the following intervention study designs:

► A 2‐arm study with an intervention group where the behavioural and environmental components cannot be separated, as compared to a control group with no intervention or business as usual;
► A multi‐arm study with: i) an experimental group with both behavioural and environmental components, ii) an experimental group with only environmental components, iii) an experimental group with only behavioural components, as compared to a control group with no intervention or business as usual.

A separate meta‐analysis will be conducted for each intervention type (single vs multi‐component), by dimension (i.e. food availability, food accessibility, food promotion, food pricing, food policy) and outcome, data permitting.

As well, food assistance programs at the household (i.e. cash transfers) or school (school feeding/meal programs) and food fortification at the industrial (i.e. mass fortification), retail and household (i.e. targeted fortification or home fortification with multiple micronutrient powders), levels are important mediators in the relationship between the food environment and diet‐related health outcomes. However, these specific pathways are outside of the scope of our review.

#### Types of interventions

Comparators include participants with no intervention, those who receive the standard of care or business as usual. We will not combine studies with business as usual and active comparison groups in the same meta‐analysis. Comparisons may be between groups of consumers/students, schools, or geographical areas such as cities (Table 2).

#### Types of outcome measures

Food environment interventions and their effect on primary and secondary outcomes will be prioritised throughout this review. Both direction of effects will be included. Our primary outcomes will be used as eligibility criteria for including studies (i.e. if a study does not report any of the primary outcomes, it will be excluded).

‐ Primary outcomes

Primary outcomes will encompass anthropometric and diet‐related health outcomes relevant to school‐age children and adolescents including (see Table 2 and Table 4 for more details):

**Table 4:** Primary and secondary outcomes

**Outcomes**	Measures
**Primary Outcomes**	
Anthropometric outcomes	Incidence, prevalence or mean difference in: ► Stunting^a^ ► Wasting^b^ ► BMI^c^, age‐ and sex‐standardized body mass index (zBMI or BMI z‐scores), overweight, obesity ► Lean mass %, fat mass % ► Waist circumference, waist‐to‐hip ratio
Diet‐related health outcomes (other than body weight)	Incidence, prevalence or mean difference in: ► Anaemia^d^ ► Type 2 diabetes^e^ ► Metabolic syndrome^f^ ► Goitre^g^ ► Xerophthalmia and Night blindness^h^
**Secondary Outcomes**	
Biomarkers of diet‐related health outcomes	Increases or decreases in the following: ► Serum ferritin ► Plasma glucose ► Urinary iodine ► Serum retinol ► Mean systolic blood pressure ► Lipid profiles
Direct measures of consumption	► Frequency of consumption ► Energy intake ► Macronutrient and micronutrient intake ► Food group intake ► Food consumption score ► Portion size
Direct measures of diet quality	► Dietary diversity score ► Meal score ► Healthy eating index or other diet quality indices ► Diet adequacy
Place of consumption	► Positive change in place of consumption which reduces risk of a diet‐related health outcome (i.e. reduced eating at fast food outlets) ► Adverse change in place of consumption which increases the risk of a diet‐related health outcome (i.e. increased eating at fast food outlets)
Meal patterns	► Positive change in meal patterns (i.e. increased fruit and vegetable intake) ► Adverse change in meal patterns (i.e. breakfast skipping)
Purchasing	► Place of purchase ► Types of food purchased by adolescents

Footnotes

a Defined as a height‐for‐age z‐score (HAZ) less than ‐2 standard deviations from the World Health Organization Growth Standards median.

b Defined as a weight‐for‐height z‐score less than ‐2 standard deviations from the World Health Organization Growth Standards median.

c BMI is defined as a simple index of weight‐for‐height that is commonly used to classify overweight and obesity in adults. It is defined as a person's weight in kilograms divided by the square of their height in meters (kg/m^2^). For children and adolescents aged 5 to 19 years, BMI‐for‐age is used. Overweight is defined as a BMI greater than or equal to 25 or weight‐for‐height z‐score greater than + 1 standard deviations from the World Health Organization Standards median, while obesity is a BMI greater than or equal to 30 or weight‐for‐height z‐score greater than + 2 standard deviations.

d Defined as a haemoglobin concentration (g/l) of 110‐119 for mild anaemia; 80‐109 for moderate anaemia; lower than 80 for severe anaemia both male and female children 5‐9.9 years and adolescents 10‐14 years, as well as non‐pregnant woman 15 years and above. In males 15 years of age and above is defined as a haemoglobin concentration (g/L) of 110‐129 for mild anaemia; 80‐109 for moderate anaemia and lower than 80 for severe anaemia).

e According to 2018 Clinical Practice Guidelines from Diabetes Canada, and World Health Organization, Type 2 diabetes is defined as a fasting plasma glucose, > 7.0 mmol/L (or 126 mg/dL).

f As defined by the International Diabetes Foundation, clinical features of metabolic syndrome in children aged 10‐16 years include obesity and 2 or more of the following criteria: fasting blood glucose > 100 mg/dL (56 mmol/L); systolic blood pressure > 130 mm Hg or diastolic blood pressure > 85 mm Hg; fasting triglycerides > 150 mg/dL; HDL < 40 mg/dL. In adolescents > 16 years, adult criteria are used, including central obesity (waist circumference) > 94 cm (men) or > 80 cm (women) and 2 of the following: fasting blood glucose > 100 mg/dL (56 mmol/L) or previously diagnosed type 2 diabetes; systolic blood pressure > 130 mm Hg or diastolic blood pressure > 85 mm Hg or treatment for hypertension; fasting triglycerides > 150 mg/dL or treatment for hyperlipidaemia; HDL < 40 mg/dL (men) or < 50 mg/dL (women) or treatment for hyperlipidaemia.

g WHO defines goitre as chronic iodine deficiency and is quantified in a population using the total goitre rate (TGR). A TGR of 5% (the number of goitres of grades 1 and 2 detected in a population divided by the number of individuals examined) or more in school‐age children (6‐12 years) is used to identify a public health problem (Vitamin and Mineral Nutrition Info Syst, 2014).

h WHO defines night blindness as serum retinol concentrations below 1.0 µmol/L. In a population this equates to a minimum prevalence of > 1% in children 6 months to 6 years (Vitamin and Mineral Nutrition Info Syst, 2014b).

1. Anthropometric outcomes (Incidence, prevalence or change in):
  ► Stunting
  ► Wasting
  ► BMI, age‐ and sex‐standardized body mass index (zBMI or BMI z‐scores), overweight, obesity
  ► Lean mass %, fat mass %
  ► Waist circumference, waist‐to‐hip ratio
2. Diet‐related health outcomes (Incidence, prevalence or change in):
  ► Anaemia
  ► Type 2 Diabetes
  ► Metabolic Syndrome
  ► Goitre
  ► Xerophthalmia and Night blindness

‐ Secondary outcomes

Secondary outcomes will surround dietary intake and biomarkers of diet‐related health outcomes including (see Table 4 for more details):

► Biomarkers of diet‐related health outcomes (e.g. Serum Ferritin, Serum Retinol, Plasma Glucose, Urinary Iodine, Mean Systolic Blood Pressure, Lipid Profiles)
► Direct measures of consumption (i.e. Frequency of Consumption, Energy Intake, Macronutrient and Micronutrient Intake, Food Group Intake, Food Consumption Score, Portion Size)
► Direct measures of diet quality (i.e. Diet Diversity Score, Meal Score, Healthy Eating Index, Other Diet Quality Indices, Diet Adequacy)
► Place of consumption (i.e. Positive Change or Adverse Change in Place of Consumption)
► Meal patterns (i.e. Positive Change or Adverse Change in Meal Patterns)
► Purchasing (Place of purchase, type(s) of food purchased)

‐ Adverse outcomes of the intervention

Food environment interventions which modify the built and natural physical environments, legal and political environments, socio‐economic, and cultural environments, may cause adverse effects in:

► Primary outcomes: increase in the prevalence or incidence of stunting or wasting; increase in the prevalence or incidence of overweight or increase in mean BMI;
► Secondary outcomes: increases in mean lipid profile or mean systolic blood pressure, changes in place of consumption (i.e. change in place of dinner consumption such as at restaurant versus at the home) or negative changes in meal patterns (i.e. breakfast skipping, snacking in place of meals or snacking in addition to meals) (Table 4).

#### Duration of follow‐up

Studies will not be excluded based on a pre‐specified minimum duration of follow‐up. However, a subgroup analysis based on follow‐up time will be conducted, depending on data availability. If studies assessed the intervention at multiple time points, we will use baseline and end of intervention time points. We will include the outcome measures most similar to those presented in the other studies included in any single meta‐analysis, and report any additional follow‐ups narratively.

#### Types of settings

We will include studies that have conducted an intervention in any LMIC setting as defined by the World Bank (2018), as long as all other inclusion criteria are met. We anticipate the majority of studies will be conducted in urban cities, at the school or community level (including out‐of‐school childcare).

The World Bank provides a comprehensive list of all the countries falling under this LMIC definition.

### Search strategy

A comprehensive search of the literature will be conducted from the year 2000 onwards, without language restrictions or search filters. According to previous systematic reviews on food environments in HIC, from 1990 to 2015, food environment research did not gain momentum until the year 2002 (McKinnon et al., 2009, Lytle and Sokol, 2017). We found this also to be true of LMICs, as our food environment scoping review did not find an eligible study prior to the year 2000.

#### Electronic searches

We will search the following academic health databases, as they cover most of the main nutrition, general health and medical journals that publish in this area:

► BiblioMap (EPPI‐Centre database of health promotion research)
► Cochrane Central Register of Controlled Trials (CENTRAL)
► Embase
► PsycINFO
► MEDLINE
► TRoPHI (EPPI‐Centre Trials Register of Promoting Health Interventions)

We will use the search strategy described in Table 5 to search MEDLINE.

**Table 5:** MEDLINE search strategy

**Search number**	**Search terms**	**Results**
**Set A**	**Food environment terms**	** **
1	(food* or nutrition*).mp	786905
2	((built or consumer or house* or home* or school* or neighbo* or communit* or work*) adj3 environment*).mp	36892
3	(bar or bars or cafe* or canteen* or cart* or caseta* or convenien* or grocer* or kiosk* or market* or outlet* or restaurant* or retail* or shop* or stall* or store* or supermarket* or take away or take‐out* or takeaway* or takeout or tuck shop* or tuckshop* or vendor* or vending).mp	545529
4	(school adj3 program*).mp	7901
5	(access* or ad or ads or advertis* or afford* or aid* or assist* or availab* or basket* or cash* or coupon* or curricul* or density or desert* or education* or exposure* or fast* or handl* or incentive* or label* or law or laws or legislat* or media* or polic* or price* or procur* or promot* or provision* or proximity or purchas* or regulat* or safe* or sale* or sell* or service* or street or supply or tax or taxes or transition* or voucher*).mp	10121997
6	foodscape*.mp	37
7	exp Child Nutritional Sciences/	476
**Set B**	**Developing countries terms**	
8	exp developing countries/	69311
9	(Africa or Asia or Caribbean Region or West Indies or South America or Latin America or Central America or Afghanistan or Albania or Algeria or Angola or Argentina or Armenia or Armenian or Azerbaijan or Bangladesh or Benin or Byelarus or Byelorussian or Belarus or Belorussian or Belorussia or Belize or Bhutan or Bolivia or Bosnia or Herzegovina or Hercegovina or Botswana or Brazil or Bulgaria or Burkina Faso or Burkina Fasso or Upper Volta or Burundi or Urundi or Cambodia or Khmer Republic or Kampuchea or Cameroon or Cameroons OR Cameron or Camerons or Cape Verde or Central African Republic or Chad or China or Colombia or Comoros or Comoro Islands or Comores or Mayotte or Congo or Zaire or Costa Rica or Cote d'Ivoire or Ivory Coast or Cuba or Djibouti or French Somaliland or Dominica or Dominican Republic or East Timor or East Timur or Timor Leste or Ecuador or Egypt or United Arab Republic or El Salvador or Eritrea or Ethiopia or Fiji or Gabon or Gabonese Republic or Gambia or Gaza or Georgia Republic or Georgian Republic or Ghana or Gold Coast or Grenada or Guatemala or Guinea or Guiana or Guyana or Haiti or Honduras or India or Maldives or Indonesia or Iran or Iraq or Isle of Man or Jamaica or Jordan or Kazakhstan or Kazakh or Kenya or Kiribati or Kosovo or Kyrgyzstan or Kirghizia or Kyrgyz Republic or Kirghiz or Kirgizstan or “Lao PDR” or Laos or Lebanon or Lesotho or Basutoland or Liberia or Libya or Macedonia or Madagascar or Malagasy Republic or Malaysia or Malaya or Malay or Sabah or Sarawak or Malawi or Nyasaland or Mali or Marshall Islands or Mauritania or Mauritius or Agalega Islands or Mexico or Micronesia or Middle East or Moldova or Moldovia or Moldovian or Mongolia or Montenegro or Morocco or Ifni or Mozambique or Myanmar or Myanma or Burma or Namibia or Nepal or Nicaragua or Niger or Nigeria or Muscat or Pakistan or Palau or Palestine or Panama or Paraguay or Peru or Philippines or Philipines or Phillipines or Phillippines or Romania or Rumania or Roumania or Russia or Russian or Rwanda or Ruanda or Saint Lucia or St Lucia or Saint Vincent or St Vincent or Grenadines or Samoa or Samoan Islands or Navigator Island or Navigator Islands or Sao Tome or Senegal or Serbia or Montenegro or Seychelles or Sierra Leone or Sri Lanka or Ceylon or Solomon Islands or Somalia or Sudan or Suriname or Surinam or Swaziland or Syria or Tajikistan or Tadzhikistan or Tadjikistan or Tadzhik or Tanzania or Thailand or Togo or Togolese Republic or Tonga or Tunisia or Turkey or Turkmenistan or Turkmen or Uganda or Ukraine or USSR or Soviet Union or Union of Soviet Socialist Republics or Uzbekistan or Uzbek or Vanuatu or New Hebrides or Venezuela or Vietnam or Viet Nam or West Bank or Yemen or Yugoslavia or Zambia or Zimbabwe or Rhodesia).mp	1428590
10	(developing countr* or “developing country” or “developing countries” or “developing nation” or “developing nations” or “developing population” or “developing populations” or “developing world” or “less developed country” or “less developed countries” or “less developed nation” or “less developed nations” or “less developed population” or “less developed populations” or “less developed world” or “lesser developed country” or “lesser developed countries” or “lesser developed nation” or “lesser developed nations” or “lesser developed population” or “lesser developed populations” or “lesser developed world” or “under developed country” or “under developed countries” or “under developed nation” or “under developed nations” or “under developed population” or “under developed populations” or “under developed world” or “underdeveloped country” or “underdeveloped countries” or “underdeveloped nation” or “underdeveloped nations” or “underdeveloped population” or “underdeveloped populations” or “underdeveloped world” or “middle income country” or “middle income countries” or “middle income nation” or “middle income nations” or “middle income population” or “middle income populations” or “low income country” or “low income countries” or “low income nation” or “low income nations” or “low income population” or “low income populations” or “lower income country” or “lower income countries” or “lower income nation” or “lower income nations” or “lower income population” or “lower income populations” or “underserved country” or “underserved countries” or “underserved nation” or “underserved nations” or “underserved population” or “underserved populations” or “underserved world” or “under served country” or “under served countries” or “under served nation” or “under served nations” or “under served population” or “under served populations” or “under served world” or “deprived country” or “deprived countries” or “deprived nation” or “deprived nations” or “deprived population” or “deprived populations” or “deprived world” or “poor country” or “poor countries” or “poor nation” or “poor nations” or “poor population” or “poor populations” or “poor world” or “poorer country” or “poorer countries” or “poorer nation” or “poorer nations” or “poorer population” or “poorer populations” or “poorer world” or “developing economy” or “developing economies” or “less developed economy” or “less developed economies” or “lesser developed economy” or “lesser developed economies” or “under developed economy” or “under developed economies” or “underdeveloped economy” or “underdeveloped economies” or “middle income economy” or “middle income economies” or “low income economy” or “low income economies” or “lower income economy” or “lower income economies” or “low gdp” or “low gnp” or “low gross domestic” or “low gross national”)or “lower gdp” or “lower gnp” or “lower gross domestic” or “lower gross national” or lmic or lmics or “third world” or “lami country” or “lami countries” or “transitional country” or “transitional countries”).mp	138364
**Set C**	**Joint Terms**	
11	1 and 2	47810
12	1 and 3	3172
13	1 and 4	1377
14	1 adj5 5	130768
15	6 or 7 or 11 or 12 or 13 or 14	163924
16	7 or 8 or 9	1483929
17	26 and 27	24846
18	17 NOT (exp animal/ not humans/)	22068
19	Limit 18=”2000‐Current”	16417

Footnotes

The search syntax has been developed for use with the Ovid search interface. The number of results provided below are from a test run conducted on February 9^th^ 2018 in the database Ovid MEDLINE(R) Epub Ahead of Print, In‐Process & Other Non‐Indexed Citations, Ovid MEDLINE(R) Daily and Ovid MEDLINE(R) 1946 to Present

We will modify this search appropriately for other databases and clinical trials registers. We will use the Ovid search interface for MEDLINE, Embase, PsycINFO and CENTRAL (see Table 3 for more details).

#### Searching other resources

Additionally, we will include unpublished data and grey literature that meet our eligibility criteria. Some of the electronic databases specified above index a combination of published and unpublished studies, such as doctoral dissertations and conference abstracts. We will not search in dissertation and abstract specific databases, as these are not considered eligible study types.

#### Unpublished studies

► International Clinical Trials Registry Platform

#### Grey literature searching

To ensure maximum coverage of unpublished literature, and reduce the potential for publication bias, we will search the following organisational websites and databases using the keyword search for unpublished grey literature in the area of health, health promotion, nutrition and the food environment including the following:

► eLENA (WHO e‐Library of Evidence for Nutrition Actions)
► FAO
► International Food Policy and Research Institute (IFPRI)
► International Initiative for Impact Evaluation (3ie)
► INFORMAS
► National Cancer Institute, Epidemiology and Genomics Research Program
► World Bank
► World Food Programme (WFP)
► World Obesity

#### Reference list searching

Reference lists of relevant systematic reviews found within our database search will be reviewed for studies that meet our eligibility criteria.

### Description of methods used in primary research

Many food environment interventions studies employ either a RCT or NRCT methodological design. Eligible study examples of each are described below:

► Safdie, M., Jennings‐Aburto, N., Lévesque, L., Janssen, I., Campirano‐Núñez, F., López‐Olmedo, N., Aburto, T. and Rivera, J. *Impact of a school‐based intervention program on obesity risk factors in Mexican children*. Salud Pública de México. 2013 55 (3): S374‐S387. This study conducted a cluster randomised controlled trial, where schools were assigned to one of three conditions: basic intervention, plus intervention or control over two years. Baseline, midpoint (7 months and 11 months) and endpoint measurements of children's height, weight and BMI were taken. The basic program focused on improving norms related to nutrition and physical activity at the schools and was limited to using existing school infrastructure and resources. The nutrition component specifically related to increasing availability of healthy food (fruits, vegetables, and non‐fried dishes) and beverages (particularly water), while reducing the availability of energy‐dense foods and sugar‐sweetened beverages, and reducing the number of eating opportunities during the school day. The plus intervention implemented all the components incorporated in the basic program and included additional financial investment and human resources. No changes were made to existing nutrition or physical activity practices in control schools.
► Fotu KF, Millar L, Kremer P, Moodie M, Snowdon W, Utter J, Vivili J, Schultz, T, Malakellis M, McCabe MP, Roberts G and Swinburn BA. *Outcome results for the Ma'alahi Youth Project, a Tongan community‐based obesity prevention programme for adolescents.* Obesity Reviews. 2011 Oct 12: Suppl.2, 41‐50. This study is a 3‐year, quasi‐experimental cohort study that examined the effects of community‐based interventions on nutritional health status in Tongan adolescents. The community‐based interventions included food environment interventions such as food policies in schools, implementation of school gardens, fruit tree planting, regulation of school canteen and a water promotion campaign. Schools located in the districts of Houma, Nukunuku and Kolonga on the main island of Tongatapu were assigned to the intervention group. Secondary schools located on the island of Vava'u were assigned to the control comparison group. Baseline anthropometric data (height, weight, waist circumference and body fat percentage) were collected in 2005‐2006. Follow up data was collected almost 3 years later in 2008.

### Criteria for determination of independent findings

As included studies will be interventional in nature and the food environment is a complex domain in which to work, we anticipate there to be heterogeneity in the literature and do not anticipate non‐independence of findings to be problematic. We will ensure that any analysis will take possible sources of dependency into consideration. If there are two or more papers describing the same study, they will be combined and coded as a single study. We will cluster papers into exposures categories and ensure that no double counting of data occurs when synthesizing across exposures.

### Details of study coding categories

After the candidate studies have been screened and the eligible studies have been selected, the two first authors (BC and CO) will code the studies following the guidelines described in the coding scheme below:

1. General information
  ► Study title (free text)
  ► Authors (free text)
  ► Year of publication (free text)
  ► Location type (single‐country LMIC, multi‐country LMIC, multi‐country HIC and LMIC)
  ► Country
  ► World Bank region
  ► World Bank income‐level (low, lower‐middle, upper‐middle, high)
  ► Study design
  ► Length of study (free text)
  ► Language
  ► Type of data (quantitative, qualitative, both)
2. Study setting and population
  ► Setting (urban, rural, peri‐urban, urban slum, mixed, other, not specified)
  ► City/Town (free text)
  ► Type of population (general, school‐age children & adolescent specific, adult, other, not specified)
  ► Context (public vs private, low SES vs high SES)
  ► Sample size reported
  ► Sample size analysed
  ► Age unit (%, mean, median, range)
  ► Age value
  ► Descriptive statistics value (free text)
  ► Descriptive statistics unit (lower confidence interval, upper confidence interval, SD, SE, P value, IQR)
  ► Gender (male, female, mixed, other, not specified)
  ► Gender value
  ► Gender unit (%, mean, median)
  As we expect variability in reported age bands, we will make a post‐hoc decision in analysis of results at different age bands. If possible, we hope to analyse data by WHO age group classifications:
  ► School‐age (5‐9.9 years)
  ► Young Adolescents (10‐14.9 years)
  ► Older Adolescents (15‐19.9 years)
3. Exposures
  ► Type of food environment (home, school, work, consumer‐mobile, consumer‐restaurant, consumer‐grocery store, other)
  ► Context (public school, private school, mixed schools, low SES, middle SES, high SES, mixed SES, undisclosed)
  ► Level collected (individual, household, community, school, market, population)
  ► Level reported (individual, household, community, school, market, population)
  ► Instruments/methodologies used (interview, checklist, questionnaire, GIS, macro food supply analysis, store audit, sales analysis)
  ► Instruments/methodologies used validated? (Yes, No, Other, NA)
  ► Instruments/methodologies used (free text and citation if available)
  ► Exposures reported domain (availability, accessibility, promotion, policy, price, other, NA)
  ► Exposures (free text)
  ► Level of Exposures (by age band, public school, private school, low SES, middle SES, high SES, by BMI status, urban, rural, peri‐urban, slum)
  ► Other exposures (free text)
  ► Study characterization of healthy/unhealthy sources of food or food products (free text)
4. Intervention
  ► Start of intervention (i.e. the date of implementation of the first intervention)
  ► End of intervention (i.e. the date of when the last intervention component was discontinued)
  ► Duration of the intervention (i.e. the time span from the start of the intervention to the end of the intervention)
  ► Total duration of the follow‐up (i.e. the time span from the start)
  ► Mode of delivery of intervention (i.e. face‐to‐face, internet, telephone)
  ► Type of intervention 1 (behavioural, environmental, both, other, not disclosed)
  ► Type of intervention 2 (preventive, management‐based, both, other, not disclosed)
  ► Who implemented the intervention? (national government, regional government, local government, school‐based, work‐based, other, not disclosed)
  ► Context of the intervention (free text)
  ► Unit of randomisation or control
  ► Unit of analysis
  ► Total number of persons in all intervention and control groups at baseline and post‐intervention
  ► Number of individuals in each intervention and control group (at baseline and at post‐intervention)
  ► If multiple time points, number of participants at each time point (attrition rate)
  ► Reasons for attrition, if provided
  ► Mode of analysis (intention‐to‐treat, per protocol, undisclosed)
  ► Which primary outcome(s) did the intervention address? (Anthropometric, diet‐related, both, other, undisclosed)
  ► Was a subgroup analysis done? (Yes or No; if yes, provide details)
  ► Adverse Outcomes or Unintended Consequence (Yes, No, Undisclosed/Unclear)
  ► General description of participants (context)
  ► Was intervention targeted at specific age categories/bands?
  ► Type of recruitment (how were participants recruited)
  ► Date of baseline assessment
  ► Date of last outcome assessment
  ► Baseline assessment of intervention group
  ► Baseline assessment of control group
  ► Any baseline differences noted between study groups?
  ► Follow‐up assessment of intervention group
  ► Follow‐up assessment of control group
  ► Post‐intervention assessment of intervention group
  ► Post‐intervention assessment of control group
  ► Measure of effect value (free text)
  ► Measure of effect (OR, RR, Rate Ratio, SMD, MD, Risk Difference, Proportion, Incidence, Prevalence)
  ► Measure of effect unit
  ► Descriptive statistics value (free text)
  ► Descriptive statistics unit ((lower confidence interval, upper confidence interval, SD, SE, P value, IQR)
5. Primary outcomes
  ► Type of primary outcome (anthropometric, diet‐related)
  ► Anthropometric or diet‐related health outcome drop‐down (BMI, Weight, Height, H/W Ratio, Waist circumference, Mid upper arm circumference (MUAC), Normal, Overweight, Obese, Overweight/Obese, Underweight, Stunting, Wasting, HAZ, BAZ, Hyperglycaemia, Diabetes Status, Iron Status, Hypertension, Vitamin A Status, Hyperlipidaemia, Other)
  ► How was outcome assessed? (tool or measurement)
  ► Is the outcome tool or measurement validated? (Yes, No, Unclear)
  ► Outcome tool used validated how and when (free text)
  ► Name of outcome tool or measurement (free text)
  ► Reference for weight outcomes (WHO cut‐offs, IOTF cut‐offs, CDC cut‐offs, Other, undisclosed))
  ► Anthropometric outcome or diet‐related health outcome unit (%, N, mean, median, z‐score, percentile, undisclosed)
  ► Anthropometric outcome 1 descriptive statistics value (free text)
  ► Anthropometric outcome 1 descriptive statistics value unit
  ► Anthropometric outcome 1 (free text)
  ► Context (how the author defined weight categories i.e. overweight vs obese)
6. Secondary outcomes
  ► Biomarker type (serum ferritin, plasma glucose, urinary iodine, serum retinol, mean systolic blood pressure, lipid profile)
  ► Biomarker other (free text)
  ► Biomarker unit (%, mean, median, mm of Hg, etc.)
  ► Biomarker descriptive statistics value (free text)
  ► Biomarker descriptive statistics value unit
  ► Frequency of consumption value (free text)
  ► Frequency of consumption unit (times/day, times/week, times/month, mL/day, mL/week, mL/month, servings/day, servings/week, servings/month, mg/day, mg/week, mg/month)
  ► Type of intake (energy, macronutrient, micronutrient, food group, other)
  ► Instrument/tools of measurement (Food frequency questionnaire [FFQ], 24‐hr recall, food diary, self‐administered questionnaire, interviewer‐assisted questionnaire, dietary recall (other than 24 hours))
  ► Validated Tool (yes, no)
  ► Name of outcome tool or measurement (free text)
  ► Food group categorization (Food and Nutrition Technical Assistance [FANTA] modified dietary diversity food groups)
  ► Energy intake/macronutrient/micronutrient reference (% of total calories from fat, % of total calories from carbohydrates, % of total calories from protein, total calories, adequate intake, estimated average requirement, recommended dietary allowance)
  ► Energy intake/macronutrient/micronutrient value (free text)
  ► Energy intake/macronutrient/micronutrient units
  ► Energy intake/macronutrient/micronutrient descriptive statistics value (free text)
  ► Energy intake/macronutrient/micronutrient descriptive statistics units
  ► Type of dietary score (food consumption score, dietary diversity score, food consumption score, health eating index etc.)
  ► Dietary score value (free text)
  ► Frequency of consumption value (free text)
  ► Frequency of consumption unit (times/day, times/week, times/month, mL/day, mL/week, mL/month, servings/day, servings/week, servings/month, mg/day, mg/week, mg/month)
  ► Place of consumption (home, school (food purchased at school), school (food brought from home), work (food bought at work), work (food brought from home), eating (purchased or home cooked food) outside of the home, eating out of the home (restaurants, food stalls etc.))
  ► Type of meal skipped (breakfast, lunch, dinner)
  ► Snacking (yes, no)
  ► Snacking context (in place of a meal, between meals, in addition to meals)
  ► Snacking frequency (times/day, times/week, times/month, mL/day, mL/week, mL/month, servings/day, servings/week, servings/month, mg/day, mg/week, mg/month)
  ► Purchasing (yes, no)
  ► Place of purchase (school, restaurant, fast‐food, street vendor, market, grocery store, other)
  ► Type of food purchased (fruits, vegetables, sweet food, salty/fried food, fast‐food, sugar‐sweetened beverages, grains/roots/tubers, pulses, nuts/seeds, condiments/seasonings, eggs, meat/poultry/fish, dairy, other)
  ► Type of food purchased (free text)
7. Other notes (Free text)

### Assessment of risk of bias in included studies

Risk of bias of included studies will be independently assessed by two review authors using the EPOC‐adapted Cochrane Collaboration ‘Risk of bias’ tool (Higgins and Green, 2011). Inconsistencies will be resolved by discussion, and where necessary by consulting a third review author. The EPOC‐adapted Cochrane ‘Risk of bias’ tool has been validated, and is commonly used in Cochrane and non‐Cochrane reviews. For controlled study designs (RCTs and NRCTs, CBAs), the assessment is based on the following seven criteria:

1. Random sequence generation (checking for possible selection bias)
  We will describe the method used to generate the allocation sequence in sufficient detail to allow an assessment of whether it produced comparable groups, for each included study. We will assess the method as:
  ► low risk of bias (any truly random process, e.g. random number table; computer random number generator);
  ► high risk of bias (any non‐random process, e.g. odd or even date of birth; hospital or clinic record number);
  ► unclear risk of bias.
2. Allocation concealment (checking for possible selection bias)
  We will describe the method used to conceal the allocation sequence in sufficient detail and determine whether intervention allocation could have been foreseen in advance of, or during recruitment, or changed after assignment, for each included study.
  We will assess the methods as:
  ► low risk of bias (e.g. telephone or central randomisation; consecutively numbered sealed opaque envelopes);
  ► high risk of bias (open random allocation; unsealed or non‐opaque envelopes, alternation; date of birth);
  ► unclear risk of bias.
3. Blinding (checking for possible performance bias)
  We will describe the methods used, if any, to blind study participants and other individuals (i.e. parents, teachers, school staff, outcome assessors) from knowledge of which intervention a participant received, for each included study. We will consider if studies were at low risk of bias if they were blinded, or if we judged that the lack of blinding would be unlikely to affect results. We will assess blinding separately for different outcomes or classes of outcomes.
  We will assess the methods as:
  ► low risk of bias (e.g. if blinding was reported of participants and personnel)
  ► high risk of bias (e.g. if blinding was not reported for participants and personnel)
  ► unclear risk of bias.
4. Blinding (checking for detection bias)
  We will describe the methods used, if any, to blind outcome assessors from knowledge of which intervention a participant received, for each included study.
  We will assess the methods as:
  ► low risk of bias (e.g. if outcome assessors were blinded)
  ► high risk of bias (e.g if outcome assessors were not blinded)
  ► unclear risk of bias.
5. Incomplete outcome data (checking for possible attrition bias through withdrawals, dropouts, protocol deviations)
  We will describe for each included study, and for each outcome or class of outcomes, the completeness of data including attrition and exclusions from the analysis. We will state whether attrition and exclusions were reported, the numbers included in the analysis at each stage (compared with the total randomised participants), reasons for attrition or exclusion where reported, and whether missing data were balanced across groups or were related to outcomes. Where sufficient information was reported, or could be supplied by the trial authors, we re‐included missing data in the analyses which we undertook. We will assess methods as:
  ► low risk of bias (e.g. no missing outcome data; missing outcome data balanced across groups);
  ► high risk of bias (e.g. numbers or reasons for missing data imbalanced across groups; ‘as treated’ analysis done with substantial departure of intervention received from that assigned at randomisation);
  ► unclear risk of bias.
6. Selective reporting bias
  We will describe each included study how we investigated the possibility of selective outcome reporting bias and what we found.
  We will assess the methods as:
  ► low risk of bias (where it was clear that all of the study's pre‐specified outcomes and all expected outcomes of interest to the review have been reported);
  ► high risk of bias (where not all the study's pre‐specified outcomes have been reported; one or more reported primary outcomes were not pre‐specified; outcomes of interest were reported incompletely and so could not be used; study failed to include results of a key outcome that would have been expected to have been reported);
  ► unclear risk of bias.
7. Other bias (checking for bias due to problems not covered by (1) to (6) above)
  We will describe each included study any important concerns we had about other possible sources of bias.
  We will assess whether each study was free of other problems that could put it at risk of bias:
  ► low risk of other bias;
  ► high risk of other bias;
  ► unclear whether there is risk of other bias.

#### Overall risk of bias

We will make explicit judgements about whether studies were at high risk of bias, according to the criteria given in the Handbook (Higgins and Green, 2011). With reference to (1) to (7) above, we will assess the likely magnitude and direction of the bias and whether we considered it was likely to impact on the findings.

For ITS study designs, we will use the following criteria to assess risk of bias:

1. Intervention independent of other changes
  ► Low risk if there are compelling arguments that the intervention occurred independently of other changes over time and the outcome was not influenced by other confounding variables/historic events during the study period;
  ► High risk if reported that the intervention was not independent of other changes in time.
2. Shape of the intervention effect pre‐specified
  ► Low risk if point of analysis is the point of intervention or a rational explanation for the shape of the intervention effect was give by the author(s);
  ► High risk if it is clear that the condition above is not met.
3. Intervention unlikely to affect data collection
  ► Low risk if reported that the intervention itself was unlikely to affect data collection;
  ► High risk if the intervention itself was likely to affect data collection.
4. Knowledge of the allocated interventions adequately prevented during the study
  ► Low risk if the authors state explicitly that the primary outcome variables were assessed blindly, or the outcomes are objective;
  ► High risk if the outcomes were not assessed blindly;
  ► Unclear risk if not specified in the paper.
5. Incomplete outcome data
  ► Low risk if missing outcome measures were unlikely to bias the results;
  ► High risk if the missing outcome data was likely to bias the results;
  ► Unclear risk if not specified in the paper.
6. Selective outcome reporting
  ► low risk of bias (where it was clear that all of the study's pre‐specified outcomes and all expected outcomes of interest to the review have been reported);
  ► high risk of bias (where not all the study's pre‐specified outcomes have been reported; one or more reported primary outcomes were not pre‐specified; outcomes of interest were reported incompletely and so could not be used; study failed to include results of a key outcome that would have been expected to have been reported);
  ► unclear risk of bias, if not specified in the paper.
7. Other bias
  ► Low risk if there is no evidence of other risk of biases.

### Synthesis procedures and conventions

#### Selection of studies

After removal of duplicate studies, we will perform a multistage screening process to select studies that meet the eligibility criteria (Table 6). Each title and abstract will be assessed by a single reviewer (BC or CO), removing those that are not relevant.

**Table 6:** Study eligibility criteria

Eligibility criteria
**Inclusion criteria**
► Low‐ or middle‐income country, according to World Bank (for multi‐country studies that include high‐income countries, data must be disaggregated)
► School‐age children (5‐9.9 years) and or adolescents (10‐19.9 years)
► Any language
► Data collected and study published 2000 or later
► Single or multi‐component food environment intervention (preventive or management‐based) implemented at the market, community or organisational level which relate to one of the following: ▷ Food availability ▷ Food accessibility (proximity, density or presence) ▷ Food promotion (prominence within shelves/placement, social media or advertising, population nutrition promotion) ▷ Food policy, regulation and compliance (food labelling/packaging, sales, marketing, taxes) ▷ Food pricing (market price or discounts)
► Anthropometric or Diet‐related Health Outcome (one of): ▷ Height, weight, MUAC, stunting, wasting, BMI, age‐ and sex‐standardized body mass index (zBMI or BMI z‐scores), lean mass %, fat mass %, waist circumference, waist‐to‐hip ratio, incidence and prevalence of overweight and obesity ► Incidence, prevalence or mean difference in anaemia, type 2 diabetes, metabolic syndrome, goitre, night blindness
► Study types/designs: ► Experimental and quasi‐experimental study designs (e.g. RCTs, non‐randomised controlled trials, controlled before‐after, interrupted time series, repeated measures study)
**Exclusion criteria**
► Studies that include critically ill or diseased participants (e.g. HIV or TB‐infected children)
► Intervention is at an individual‐level (i.e. food safety, food fortification, supplementation, dietary intake or physical activity‐based interventions alone)

In the second step, two review authors (BC and CO) will assess all full texts in duplicate. Any disagreements will be resolved by discussion and, where necessary, by consulting a third review author. At this stage of the screening process, we will document the reasons for exclusion. We will exclude studies without primary outcomes. We will not exclude studies on the basis of reporting of an outcome (i.e., that an effect size is not estimable although the outcome was clearly measured). We will also contact the primary authors of the study for any insufficient or unclear information if required.

EndNote will be used to collect and de‐duplicate studies and to initially screen title and abstracts. For full‐texts review and to document the exclusion decisions made, we will use the web‐based software application Covidence. Covidence has been developed to streamline the screening of literature and writing of systematic reviews. Any post‐hoc changes to the eligibility criteria that may occur at the data extraction stage will be reported explicitly in the final review.

#### Data extraction and management

Data from the included studies will be independently coded and extracted by two review authors using the predefined data extraction forms. Data extraction forms will be matched. A third review author will be consulted in the event of any disagreements, and consensus will be sought. We will attempt to contact authors of the original reports to obtain further details if required.

#### Measures of treatment effect

For dichotomous outcomes (e.g. rate of new‐onset obesity) results will be expressed as risk ratio (RR) with 95% confidence intervals (CI). Where continuous scales of measurement are used to assess the effects of a food environment exposure (e.g. consumption of fast food in times per week/per person or average BMI), the mean difference (MD) will be used, or the standardized mean difference (SMD) if different scales have been used in the primary studies. It is expected that most outcome measures will be continuous. We will include 95% CIs for MD, SMD and RR intervention effects.

#### Unit of analysis issues

We will give special attention to the unit of randomisation, unit of analysis and underlying design issues for each included study, particularly identifying those studies with cross‐over and clustered designs. We will assess whether or not analysis and reporting were appropriately done. We will make decisions about whether or not to include studies with potential errors in analysis by consensus with the review authors, and consultation with a statistician where necessary.

We will use the following questions to guide our decision:

1. What is the unit of analysis issue?
2. Can the issue be corrected by the review authors (for example, the Cochrane Handbook for Systematic Reviews of Interventions gives some guidance to review authors if a study will be included in a meta‐analysis and the original analysis did not adequately adjust for clustering).
3. Does the unit of analysis issue impact all aspects of the results and analysis or only parts of it?
4. Does the unit of analysis issue represent an error that would warrant exclusion if all other aspects of the study indicated inclusion? Could some aspects of the study be used?

Special attention will be given to cluster RCTs and cluster NRCTs, especially where analyses are not adjusted for clustering. We will attempt to adjust results for clustering according to the Cochrane Handbook (sections 16.3.4, 16.3.5 and 16.3.6. If we are unable to adjust such results, we will present these data in a separate table. In contrast, cluster RCTs that have adjusted for clustering in their analysis can be combined with RCTs randomised by individuals.

#### Dealing with missing data

In case that missing data on study characteristics or outcome measures precludes study inclusion or limits the use of a study at further stages of the review, we will contact the corresponding author. Missing data may include standard deviations for continuous outcome measures, sample sizes, standard errors or follow‐up times (Higgins and Green, 2011). For registered but unpublished trials, we will contact the corresponding investigator to request relevant data.

#### Assessment of heterogeneity

We will generate τ^2^, or Tau^2^ to estimate the absolute value of true heterogeneity between studies, and Chi² test to calculate the P value for the heterogeneity. We will consider it statistically significant if the P value for the Chi^2^ test is < 0.1.

If we identify substantial heterogeneity, we will report this and explore possible causes in pre‐specified subgroup analyses.

#### Assessment of reporting biases

To assess the potential for publication bias, we will compare mean effect sizes of published studies with those from unpublished sources. If the number of eligible studies is sufficient (recommended minimum of ten studies), funnel plots will be inspected to assess whether estimates from individual studies are symmetrically distributed around the mean effect size. As well, we will critically assess study bias and outcomes based on funding source (i.e. corporate funding versus studies with independent funding). Depending on data availability, we will conduct a sensitivity analysis comparing mean effect sizes from both types of funding.

#### Data synthesis

We will present the synthesis of quantitative evidence from the included studies through narrative and statistical analysis of comparable interventions/outcomes using meta‐analysis. Meta‐analysis is useful in synthesizing quantitative evidence from multiple studies as it considers the statistical power of the effect estimated in these various studies. We will first categorise the studies based on type of study design (i.e. RCT vs quasi‐experimental), and then by intervention type, since we will consider both single and multi‐component interventions and outcomes in our systematic review. For multi‐component interventions, we will determine which arms are most relevant to our review objectives and meet our inclusion criteria for meta‐analyses purposes, though descriptions of all intervention arms per study will be presented in a ‘Characteristics of Included Studies’ table. For example, in a three‐armed RCT (1. food environment only intervention, 2. food environment plus behavioural intervention 3. no treatment), we will only include arms 1 and 3 in our analysis. However, in a two‐armed multi‐component RCT (1. food environment plus behavioural intervention, 2. no treatment) we will analyse these studies separately from two armed single component RCT (1. food environment alone, 2. no treatment).

Depending on the outcome, we will calculate the standardised mean difference (for continuous outcomes) or risks ratio (for dichotomous outcomes), which are appropriate for comparison between groups across similar types of treatment effects. Where multiple measures are reported for an outcome in a single study, we will utilize the most commonly reported measure across all included studies. We will attempt to pool all studies within a given study design and or intervention category, assessing the same outcome, in the same population group by conducting a random‐effects meta‐analysis using Review Manager 5.3. We will generate τ^2^, or Tau^2^ to estimate the absolute value of true heterogeneity between studies. Moreover, to ensure no unit‐of‐analysis errors are made, we will combine groups to create a single pair‐wise comparisons for each meta‐analysis. Additionally, studies that include both school‐age children and adolescents, but do not disaggregate outcome data will be analysed as a separate group (i.e. 5 to 12 years). We do not plan on conducting any moderator analyses, as we are not comparing effects for different types of food environment domains (i.e. pricing vs promotion). Where meta‐analysis is deemed inappropriate due to substantial heterogeneity, we will summarise the findings of the included studies narratively.

#### Subgroup analysis and investigation of heterogeneity

A random‐effects meta‐analysis model will be used given the high level of variation in effect sizes anticipated across studies due to the wide diversity in the design, scale, and implementation of their interventions. Therefore, we will apply the random‐effects model in conducting the subgroup analyses including all between and within subgroup weighting for the specified characteristics below. With this approach, we will estimate and report the variance in effect size across the studies within each subgroup and pool these subgroup estimates based on the assumption that the between study variance is likely to be the same for all subgroups. Combined mean effect estimates of the subgroups will be computed based on the random‐effects weights and pooled tau2. Comparisons for statistically significant differences will be tested using an analysis of variance Q statistic that follows a chi‐square distribution.

From our scoping review, we have identified subgroup analyses that would be most appropriate to investigate heterogeneity (if data permits):

► Age group (school‐age vs adolescents)
► Geographical location (urban, rural, peri‐urban)
► Duration of the intervention and follow‐up (i.e. 3 to 6 months, > 6 months to 1 year, > 1 year to < 2 years, and 2 years and beyond).
► Randomization

#### Sensitivity analysis

We will conduct a comparative analysis to test for sensitivity of the results of the review by:

► comparing results if we include studies into our predetermined age bands that may have been pooled separately due to age range of participants (for example, taking the mean of a study population that includes 5‐12 year olds, and adding it to our school‐age group of studies);
► determining whether results differ when studies at high risk of bias are excluded (from any dimension)

### Treatment of qualitative research

We do not plan to include qualitative research.

### Quality of evidence

We will use the Grading of Recommendations Assessment, Development and Evaluation (GRADE) system for grading the body of evidence for outcomes. In the context of GRADE, the quality of a body of evidence is understood as the extent to which one can be confident that an estimate of an effect is close to the true effect (Guyatt et al., 2008; Schunemann et al., 2008). The assessment will be summarised with a ‘Summary of findings’ table created with the GRADE pro software. Within the GRADE approach, the quality of a body of evidence is assessed based on the design of the underlying studies and on a number of factors that can decrease or increase the quality of evidence irrespective of study design. The four possible quality ratings are shown in Table 7.

**Table 7:** Quality of evidence, as determined by GRADE criteria

**Quality**	Description
**Very low**	Any estimate of effect is uncertain.
**Low**	Further research is very likely to have an important impact on our confidence in the estimate of effect and is likely to change the estimate.
**Moderate**	Further research is likely to have an important impact on our confidence in the estimate of effect and may change the estimate.
**High**	Further research is very unlikely to change our confidence in the estimate of effect.

There are five factors that can lead to a downgrading of the level of evidence. If one of these factors is found to exist, it is classified either as serious (downgrading by one level) or as very serious (downgrading by two levels):

► Risk of bias of individual studies by dimension (limitations in the design and implementation of available studies suggesting high likelihood of bias)
► Indirectness of evidence (indirect population, intervention, control, outcomes)
► Unexplained heterogeneity or inconsistency of results (including problems with subgroup analyses)
► Imprecision of results (wide confidence intervals)
► High probability of publication bias

Moreover, there are three factors that can increase the quality of evidence, particularly in relation to quasi‐experimental studies including:

► Large effects (i.e. a relative risk reduction of 50% or more, upgrades the evidence by one level; relative risk reduction of 80% or more, upgrade by 2 levels)
► Dose response relationships
► If all plausible residual confounding & biases (would have either reduced the demonstrated effect or increased the effect if no effect was observed)

## Review authors

**Lead review author:**

**Table:** 

Name: Bianca Carducci	
Title: Clinical Research Project Coordinator	
Affiliation: The Hospital for Sick Children, The Centre for Global Child Health	
Address: 686 Bay Street	
City, State, Province or County: Toronto, Ontario	
Post code: M5G 0A4	
Country: Canada	
Phone: 416‐813‐7654 ext. 309515	
Email: bianca.carducci@sickkids.ca	
**Co‐author(s):**	
Name: Christina Oh	
Title: Clinical Research Project Assistant	
Affiliation: The Hospital for Sick Children, The Centre for Global Child Health	
Address: 686 Bay Street	
City, State, Province or County: Toronto, Ontario	
Post code: M5G 0A4	
Country: Canada	
Phone: 416‐813‐7654 ext. 301774	
Email: christina.oh@sickkids.ca	
	
Name: Emily C. Keats	
Title: Research Associate	
Affiliation: The Hospital for Sick Children, The Centre for Global Child Health	
Address: 686 Bay Street	
City, State, Province or County: Toronto, Ontario	
Post code: M5G 0A4	
Country: Canada	
Phone: 416‐813‐7654 ext. 309518	
Email: emily.keats@sickkids.ca	
	
Name: Michelle F. Gaffey	
Title: Senior Research Manager	
Affiliation: The Hospital for Sick Children, The Centre for Global Child Health	
Address: 686 Bay Street	
City, State, Province or County: Toronto, Ontario	
Post code: M5G 0A4	
Country: Canada	
Phone: 416‐813‐7654 ext. 309108	
Email: michelle.gaffey@sickkids.ca	
	
Name: Daniel Roth	
Title: Scientist and Assistant Professor	
Affiliation: The Hospital for Sick Children, The Centre for Global Child Health The University of Toronto, Department of Paediatrics & Department of Nutritional Sciences	
Address: 686 Bay Street	
City, State, Province or County: Toronto, Ontario	
Post code: M5G 0A4	
Country: Canada	
Phone: 416‐813‐7654 ext. 328807	
Email: daniel.roth@sickkids.ca	
	
Name: Zulfiqar A. Bhutta	
Title: Co‐Director and Professor	
Affiliation: The Hospital for Sick Children, The Centre for Global Child Health; The University of Toronto, Department of Nutritional Sciences & Dalla Lana School of Public Health	
Address: 686 Bay Street	
City, State, Province or County: Toronto, Ontario	
Post code: M5G 0A4	
Country: Canada	
Phone: 416‐813‐7654 ext. 328532	
Email: zulfiqar.bhutta@sickkids.ca	

## Roles and responsibilities

Content: BC, CO, EK, DR and ZAB have content expertise in the area of global child health and nutrition.

Systematic review methods: EK, MFG, DR and ZAB have expertise and experience in conducting systematic reviews and meta‐analyses.

Statistical analysis: EK, MFG, DR and ZAB have expertise in statistical methods, particularly those methods used in clinical research and epidemiology.

Information retrieval: BC, CO, and EK have expertise and experience in retrieving and abstracting information from the literature using systematic methods.

## Sources of support

Funding was received from The Joannah and Brian Lawson Centre for Child Nutrition, The University of Toronto.

## Declarations of interest

None to declare.

## Preliminary timeframe

Review submission: March 2019

## Plans for updating the review

We intend on updating the review in three years.

## AUTHOR DECLARATION

### Authors' responsibilities

By completing this form, you accept responsibility for preparing, maintaining and updating the review in accordance with Campbell Collaboration policy. Campbell will provide as much support as possible to assist with the preparation of the review.

A draft review must be submitted to the relevant Coordinating Group within two years of protocol publication. If drafts are not submitted before the agreed deadlines, or if we are unable to contact you for an extended period, the relevant Coordinating Group has the right to de‐register the title or transfer the title to alternative authors. The Coordinating Group also has the right to de‐register or transfer the title if it does not meet the standards of the Coordinating Group and/or Campbell.

You accept responsibility for maintaining the review in light of new evidence, comments and criticisms, and other developments, and updating the review at least once every five years, or, if requested, transferring responsibility for maintaining the review to others as agreed with the Coordinating Group.

### Publication in the Campbell Library

The support of the Coordinating Group in preparing your review is conditional upon your agreement to publish the protocol, finished review, and subsequent updates in the Campbell Library. Campbell places no restrictions on publication of the findings of a Campbell systematic review in a more abbreviated form as a journal article either before or after the publication of the monograph version in Campbell Systematic Reviews. Some journals, however, have restrictions that preclude publication of findings that have been, or will be, reported elsewhere and authors considering publication in such a journal should be aware of possible conflict with publication of the monograph version in Campbell Systematic Reviews. Publication in a journal after publication or in press status in Campbell Systematic Reviews should acknowledge the Campbell version and include a citation to it. Note that systematic reviews published in Campbell Systematic Reviews and co‐registered with Cochrane may have additional requirements or restrictions for co‐publication. Review authors accept responsibility for meeting any co‐publication requirements.

**I understand the commitment required to undertake a Campbell review, and agree to publish in the Campbell Library. Signed on behalf of the authors**:

**Table:** 

**Form completed by: Bianca Carducci**	**Date: 10 October 2018**
